# Supplementary material for: Identification of SLC3A2 as a Potential Therapeutic Target of Osteoarthritis Involved in Ferroptosis by Integrating Bioinformatics, Clinical Factors and Experiments
Source: Cells. 2022 Oct 30;11(21):3430. doi: 10.3390/cells11213430 (PMC9657506; doi:10.3390/cells11213430)
Supplement: Supplementary file 1 [file cells-11-03430-s001.zip › Supplementary Figure S1.pdf]

Figure captions:

Figure S1: (a) Common genes were visualized by heatmap. **(b)** A total of 54 DEFRGs were obtained. **(c)** PPI network of DEFRGs. **(d)** Screening hub genes of 10 algorithms with R package "UpSetR". **(e)** Cluster dendrogram for 38 samples and OA trait. **(f)** Soft-threshold power for WGCNA. The red line in the left panel indicates R^2 =0.85. **(g)** Clustering dendrogram for modules. **(h)** Correlation between OA trait and modules. **(i)** Two genes were obtained by WGCNA and PPI analyses. **(j)** The differential expression and diagnostic effectiveness of SLC3A2 were further validated with another GEO dataset GSE169077.

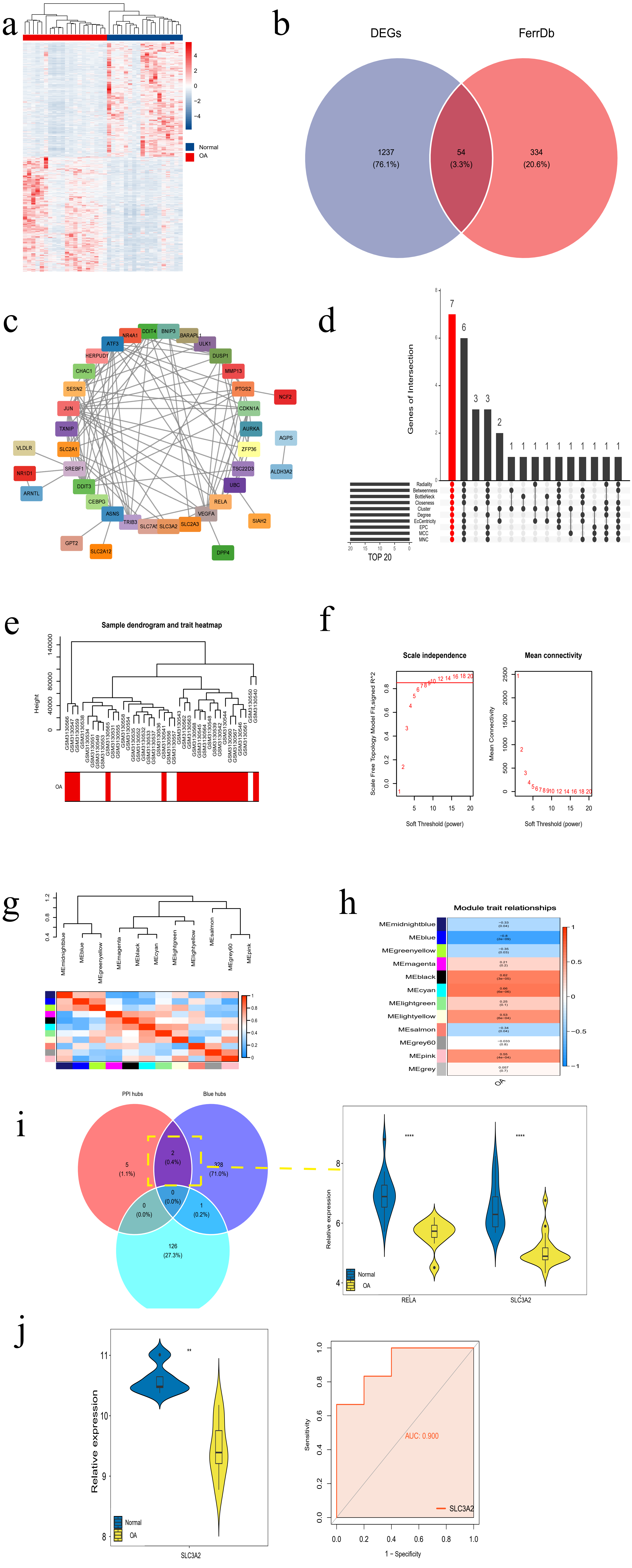

a
Normal
OA
b
DEGs
FerrDb
1237
(76.1%)
54
(3.3%)
334
(20.6%)
c
NR4A1
DDIT4
BNIP3
BARAPL1
ATF3
ULK1
HERPUD1
DUSP1
CHAC1
MMP13
SESN2
PTGS2
NCF2
JUN
CDKN1A
TXNIP
AURKA
AGPS
VLDLR
SLC2A1
ZFP36
NR1D1
SREBF1
TSC22D3
ALDH3A2
ARNTL
DDIT3
UBC
CEBPG
RELA
ASNS
VEGFA
TRIB3
SLC3A2
SLC2A3
SIAH2
GPT2
SLC2A12
DPP4
d
Genes of Intersection
Radiality
Betweenness
BottleNeck
Closeness
Cluster
Degree
EcCentricity
EPC
MCC
MNC
TOP 20
e
Sample dendrogram and trait heatmap
Height
OA
f
Scale independence
Mean connectivity
Scale Free Topology Model Fit,signed R^2
Mean Connectivity
Soft Threshold (power)
g
MEmidnightblue
MEblue
MEgreenyellow
MEmagenta
MEblack
MEcyan
MElightgreen
MElightyellow
MEsalmon
MEgrey60
MEpink
h
Module trait relationships
MEmidnightblue
MEblue
MEgreenyellow
MEmagenta
MEblack
MEcyan
MElightgreen
MElightyellow
MEsalmon
MEgrey60
MEpink
MEgrey
OA
i
PPI hubs
Blue hubs
5
(1.1%)
2
(0.4%)
328
(71.0%)
0
(0.0%)
126
(27.3%)
1
(0.2%)
Relative expression
Normal
OA
RELA
SLC3A2
j
Relative expression
Normal
OA
SLC3A2
Sensitivity
1 − Specificity
AUC: 0.900
SLC3A2
