# Supplementary material for: Identification of SLC3A2 as a Potential Therapeutic Target of Osteoarthritis Involved in Ferroptosis by Integrating Bioinformatics, Clinical Factors and Experiments
Source: Cells. 2022 Oct 30;11(21):3430. doi: 10.3390/cells11213430 (PMC9657506; doi:10.3390/cells11213430)
Supplement: Supplementary file 1 [file cells-11-03430-s001.zip › Supplementary figure S2.pdf]

Figure captions:

Figure S2: Potential association between SLC3A2, ferroptosis, and osteoarthritis in pre-clinical and clinical settings.

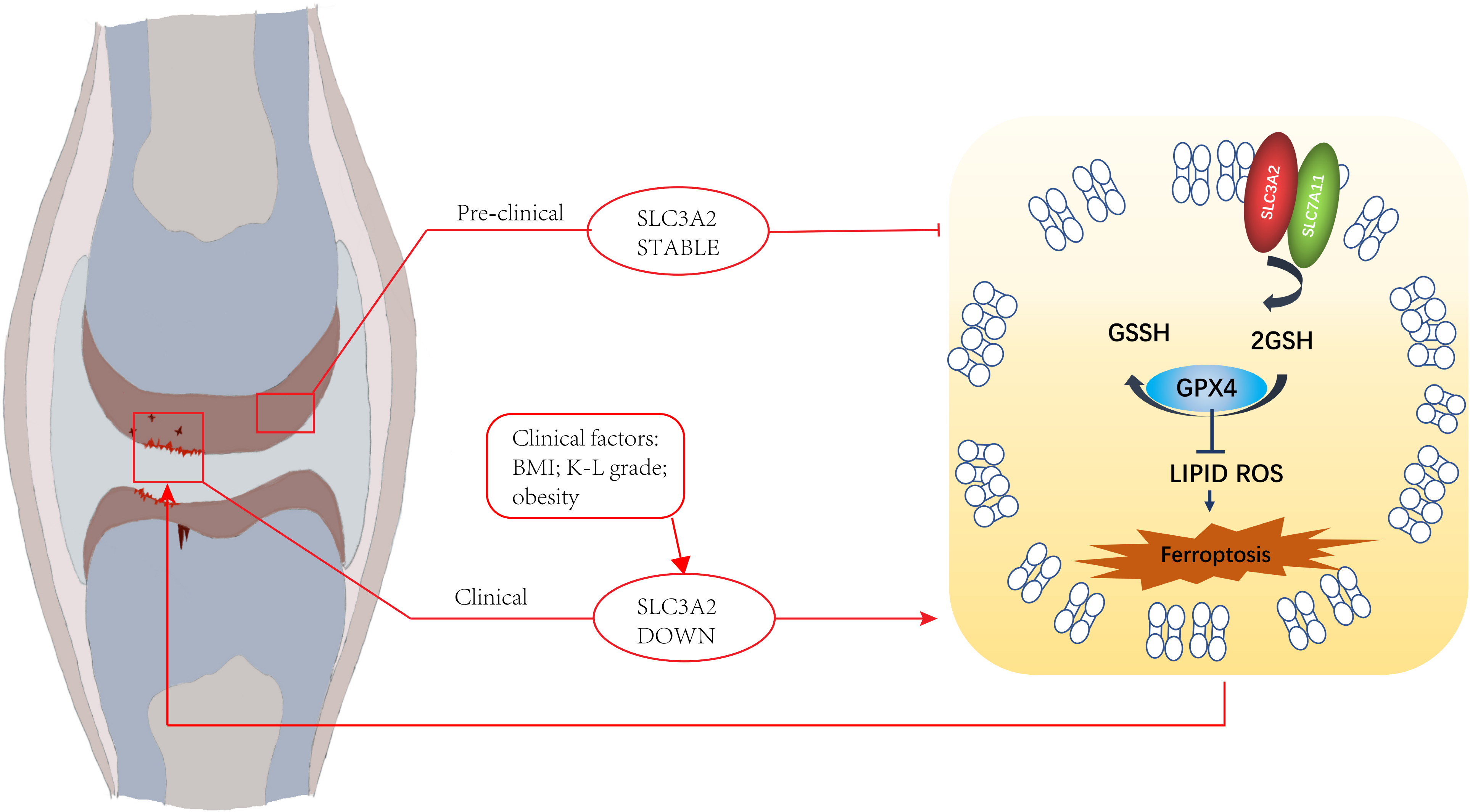

Pre-clinical
SLC3A2
STABLE
Clinical factors:
BMI; K-L grade;
obesity
Clinical
SLC3A2
DOWN
SLC3A2
SLC7A11
GSSH
2GSH
GPX4
LIPID ROS
Ferroptosis
