# Supplementary material for: Identification of SLC3A2 as a Potential Therapeutic Target of Osteoarthritis Involved in Ferroptosis by Integrating Bioinformatics, Clinical Factors and Experiments
Source: Cells. 2022 Oct 30;11(21):3430. doi: 10.3390/cells11213430 (PMC9657506; doi:10.3390/cells11213430)
Supplement: Supplementary file 1 [file cells-11-03430-s001.zip › Supplementary Table S1.pdf]

Supplementary Table 1. Basic information of GEO datasets

GEO	Type	Platform	Tissue	Samples			Country
				Normal	OA	Total	
GSE114007	RNA-seq	GPL11154<br>GPL18573	Cartilage	18	20	38	USA
GSE169077	Array	GPL96	Cartilage	5	6	11	USA
